# Gut Microbiome Associated With Graves Disease and Graves Orbitopathy: The INDIGO Multicenter European Study

**DOI:** 10.1210/clinem/dgad030

**Published:** 2023-01-23

**Authors:** Filippo Biscarini, Giulia Masetti, Ilaria Muller, Hedda Luise Verhasselt, Danila Covelli, Giuseppe Colucci, Lei Zhang, Mohd Shazli Draman, Onyebuchi Okosieme, Pete Taylor, Chantal Daumerie, Maria-Cristina Burlacu, Michele Marinò, Daniel George Ezra, Petros Perros, Sue Plummer, Anja Eckstein, Mario Salvi, Julian R Marchesi, Marian Ludgate

**Affiliations:** Division of Infection & Immunity, School of Medicine, Cardiff University, Cardiff, CF14 4XW, UK; Department of Bioinformatics, Parco Tecnologico Padano Srl (PTP), Lodi, 26900, Italy; Institute of Agricultural Biology and Biotechnology, Italian National Research Council (CNR), Milan, 20133, Italy; Division of Infection & Immunity, School of Medicine, Cardiff University, Cardiff, CF14 4XW, UK; Department of Bioinformatics, Parco Tecnologico Padano Srl (PTP), Lodi, 26900, Italy; Department of Clinical Sciences and Community Health, University of Milan, Milan, 35-I-20122, Italy; Graves' Orbitopathy Center, Endocrinology, Fondazione IRCCS Ca' Granda Ospedale Maggiore Policlinico Milan, Milan, 35-I-20122, Italy; Institute of Medical Microbiology, University Hospital Essen, University of Duisburg-Essen, Essen, 45147, Germany; Cultech Ltd., Baglan, Port Talbot, SA12 7BZ, UK; Department of Bioinformatics, Parco Tecnologico Padano Srl (PTP), Lodi, 26900, Italy; Graves' Orbitopathy Center, Endocrinology, Fondazione IRCCS Ca' Granda Ospedale Maggiore Policlinico Milan, Milan, 35-I-20122, Italy; Cultech Ltd., Baglan, Port Talbot, SA12 7BZ, UK; Graves' Orbitopathy Center, Endocrinology, Fondazione IRCCS Ca' Granda Ospedale Maggiore Policlinico Milan, Milan, 35-I-20122, Italy; Division of Infection & Immunity, School of Medicine, Cardiff University, Cardiff, CF14 4XW, UK; Centre for Stem Cell Biology, School of Biosciences, University of Sheffield, Sheffield, S10 2TN, UK; Division of Infection & Immunity, School of Medicine, Cardiff University, Cardiff, CF14 4XW, UK; KPJ Healthcare University College, Kota Seriemas, 71800 Nilai, Negeri Sembilan, Malaysia; Division of Infection & Immunity, School of Medicine, Cardiff University, Cardiff, CF14 4XW, UK; Division of Infection & Immunity, School of Medicine, Cardiff University, Cardiff, CF14 4XW, UK; Department of Endocrinology, Cliniques Universitaires Saint-Luc, Université Catholique de Louvain, Brussels, B-1200, Belgium; Department of Endocrinology, Cliniques Universitaires Saint-Luc, Université Catholique de Louvain, Brussels, B-1200, Belgium; Department of Endocrinology, University Hospital of Pisa, Pisa, 56124, Italy; Department of Clinical and Experimental Medicine, Endocrinology Unit I, University of Pisa, Pisa, 56124, Italy; Moorfields Eye Hospital NIHR Biomedical Research Centre for Ophthalmology, London and UCL Institute of Ophthalmology, London, EC4 9EL, UK; Department of Endocrinology, Royal Victoria Infirmary, Newcastle upon Tyne, NE1 4LP, UK; Cultech Ltd., Baglan, Port Talbot, SA12 7BZ, UK; Department of Ophthalmology, University Hospital Essen, University of Duisburg-Essen, Essen, 45147, Germany; Graves' Orbitopathy Center, Endocrinology, Fondazione IRCCS Ca' Granda Ospedale Maggiore Policlinico Milan, Milan, 35-I-20122, Italy; Microbiomes, Microbes and Informatics Group, School of Biosciences, Cardiff University, Cardiff, CF10 3AX, UK; Department of Metabolism, Digestion and Reproduction, Imperial College London, London, W2 1NY, UK; Division of Infection & Immunity, School of Medicine, Cardiff University, Cardiff, CF14 4XW, UK

**Keywords:** Graves disease, Graves orbitopathy, gut microbiota, autoimmunity, hyperthyroidism, *Firmicutes*:*Bacteroidetes* ratio

## Abstract

**Context:**

Gut bacteria can influence host immune responses but little is known about their role in tolerance-loss mechanisms in Graves disease (GD; hyperthyroidism caused by autoantibodies, TRAb, to the thyrotropin receptor, TSHR) and its progression to Graves orbitopathy (GO).

**Objective:**

This work aimed to compare the fecal microbiota in GD patients, with GO of varying severity, and healthy controls (HCs).

**Methods:**

Patients were recruited from 4 European countries (105 GD patients, 41 HCs) for an observational study with cross-sectional and longitudinal components.

**Results:**

At recruitment, when patients were hyperthyroid and TRAb positive, *Actinobacteria* were significantly increased and *Bacteroidetes* significantly decreased in GD/GO compared with HCs. The *Firmicutes* to *Bacteroidetes* (F:B) ratio was significantly higher in GD/GO than in HCs. Differential abundance of 15 genera was observed in patients, being most skewed in mild GO. *Bacteroides* displayed positive and negative correlations with TSH and free thyroxine, respectively, and was also significantly associated with smoking in GO; smoking is a risk factor for GO but not GD. Longitudinal analyses revealed that the presence of certain bacteria (*Clostridiales*) at diagnosis correlated with the persistence of TRAb more than 200 days after commencing antithyroid drug treatment.

**Conclusion:**

The increased F:B ratio observed in GD/GO mirrors our finding in a murine model comparing TSHR-immunized with control mice. We defined a microbiome signature and identified changes associated with autoimmunity as distinct from those due to hyperthyroidism. Persistence of TRAb is predictive of relapse; identification of these patients at diagnosis, via their microbiome, could improve management with potential to eradicate *Clostridiales*.

Graves disease (GD) affects 0.2% to 1.3% of the global population and is the most common cause of hyperthyroidism and the result of autoantibodies (TRAbs) to the thyrotropin receptor (TSHR). About 20% to 40% of GD patients develop Graves orbitopathy (GO), an autoimmune condition in which orbital tissue remodeling may produce proptosis, double vision, and risk of sight loss, although GO is mild in most cases (1). GD predominates in women (8 women:1 man) but sex bias is lower in GO, with men tending to have more severe disease.

The pathogenesis of GD/GO comprises genetic and environmental factors, with smoking being identified as a major risk element (2). A role for the gut microbiota in autoimmunity has also been proposed (3), possibly by modulating the differentiation of immature T cells in the gut-associated lymphoid tissue (4–6). This immune modulation can alter the proportion of several T-cell subtypes known to be implicated in GD/GO, such as regulatory T (Treg) and T-helper 17 (Th17) cells (7, 8), and be related to an imbalance in commensal bacteria, rather than to a single-species pathogen (9).

Our previous studies, using a TSHR-induced murine model of GD/GO, identified disease-associated taxa (eg, reduced *Bacteroides* spp in diseased mice) and correlations with disease features in BALB/c mice (10). C57/BL6 mice have a different H2 haplotype and microbiota composition and are resistant to disease induction (11). Manipulation of the BALB/c gut microbiota before disease induction proved a functional role of the gut microbiota in pathogenesis. Long-term antibiotic treatment decreased microbial diversity and reduced incidence/severity both of GD and GO, whereas fecal material transplant from severe GO increased the severity of GD but not GO (12). In humans with autoimmune thyroid diseases, bowel discomfort is often reported, varying from constipation in Hashimoto thyroiditis (ie, autoimmune hypothyroidism) to diarrhea in GD (13), considered mainly to be a consequence of thyroid dysfunction.

Previous cross-sectional studies reported perturbations in the gut microbiota in Hashimoto thyroiditis patients compared to matched healthy controls (HCs) (14, 15), and several have addressed the composition of the gut microbiota in Asian cohorts of GD and GO patients (9, 16–19) . Investigations to date have been single center and comprised small numbers of participants.

The aim of the INDIGO study (Investigation of Novel biomarkers and Definition of the role of the microbiome In Graves’ Orbitopathy) was to compare the fecal microbiota in GD patients, with GO of varying severity, and HCs. To achieve this, we applied 16S ribosomal RNA (rRNA) sequencing to analyze the gut microbiota composition from GD patients with and without GO of varying severity, from 4 European countries, and compared it with that of age- and sex-matched controls. We aimed to identify changes associated with thyroid status, eye-disease severity and related to GD/GO risk factors such as sex and smoking, using cross-sectional analyses at recruitment. We also aimed to explore the presence and persistence of species, in longitudinal analyses at more than 200 days following antithyroid drug therapy (ATD), in patients who remained TRAb positive compared with those who responded fully to treatment and became TRAb negative.

## Materials and Methods

### Study Design

We conducted an observational study that comprised both cross-sectional and longitudinal components. Cross-sectional analyses were undertaken at recruitment and comprised the majority of the eligible cohort, that is, those providing all necessary samples and clinical data. The longitudinal aspect was restricted to patients recruited early in the study who had received ATD therapy for enough time to affect both thyroid status and TRAb levels.

### INDIGO Inclusion Criteria and Ethics Approvals

Samples used in this study were collected between October 2014 and June 2016 within the framework of the EU-FP7 INDIGO project from 4 European countries and 8 centers: United Kingdom (Cardiff, Merthyr Tydfil, Newcastle Upon Tyne, and Moorfields), Italy (Milan and Pisa), Belgium (Brussels), and Germany (Essen). Appropriate local research ethics approval was obtained from all recruitment centers (Essen: Ethics Committee of the Medical Faculty of the University of Duisburg-Essen reference 14-5965-BO; Cardiff and UK centers: Wales Research Ethics reference 12/WA/0285; Milan: Comitato Etico Milano Area B, approval obtained November 11, 2014; Brussels: 2015/05JAN/002 approval obtained by Comitè d’Ethique Hospitalo-Facultaire Saint-Luc-UCL; Newcastle Upon Tyne: approval obtained from Integrated Research Application System [IRAS] reference 95037). Written informed consent was obtained from each participant at the moment of enrollment in the study. The study was conducted following the principles of the Declaration of Helsinki. Inclusion criteria were (i) GD patients untreated or at maximum of 6 weeks from commencing ATD treatment from a new diagnosis or disease relapse, (ii) euthyroid GO patients, and (iii) newly diagnosed GD patients with overt GO. Patients were excluded if they had taken any antibiotics in the 3 months before recruitment.

### Participant Assessment, Recruitment and Sample Collection

A total of 211 patients and 46 healthy controls were initially enrolled in the INDIGO study. Diagnosis was made by consultants in each recruiting center based on the following inclusion criteria: untreated Graves hyperthyroidism (or within 4 weeks of initiating ATD treatment); hyperthyroidism defined as TSH decreased, free thyroxine (FT4), and/or free triiodothyronine (FT3) increased; Graves defined as diffusely enlarged thyroid gland either by palpation or echography, and/or homogeneous thyroid uptake at scintigraphy, or positive TRAb; first episode or recurrence of Graves hyperthyroidism; GO defined as “mild,” “moderate-severe,” or “sight-threatening” eye disease, based on the EUGOGO (European Group on Graves Orbitopathy) guidelines (20). “Subclinical hyperthyroid” patients (ie, suppressed TSH but normal FT4 and/or FT3) were considered as “hyperthyroid patients.” “Euthyroid patients” were defined by FT4 thyroid hormone levels being in the normal range. HCs from each recruitment center, matched by age and sex, had not received antibiotics within at least 4 weeks of recruitment, and were all free of thyroid and eye diseases, euthyroid, and negative for TRAbs.

Apart from the lack of a fecal sample at enrollment, exclusion criteria included: (i) previous or planned treatment with ^131^I or thyroidectomy; (ii) sight-threatening GO requiring decompression procedures; (iii) drugs interfering with the natural course of GO (steroids, immunosuppressants, thiazolidinediones (20), antibiotics, antifungals, antivirals— topical as well as systemic for at least 4 weeks before recruitment to the study); (iv) acute diarrhea illness (gastroenteritis, for at least 4 weeks before recruitment to the study); (v) drugs interfering with thyroid function (amiodarone, lithium, iodine supplements); (vi) drug or alcohol abuse; (vii) no informed consent; (viii) age younger than 18 years; and (ix) pregnancy.

Microbiome analysis at enrollment (T0) was conducted on 105 patients (GD and GO) and 41 HCs.

Blood and fecal samples were obtained at T0, at the second visit, which corresponded to the first time that euthyroidism was apparent (30-252 days, median 93 days from enrollment, n = 35), and at the end of follow-up (189-644 days, median 470 days from enrollment, n = 30). (Supplementary Fig. S1A (21)). To facilitate the collection of the fecal sample at home, patients were provided a packaged kit including the correct instructions for sampling, a sterile collection tube. and a transport tube to be returned frozen to the clinic, where samples were stored frozen at −20 °C until processing. Samples at T0 and between 218 and 644 (median 471) days (ie, 200+ days; T1) were used for the longitudinal analysis (N = 23). At T1, 11 of 20 patients remained TRAb positive whereas 12 of 20 became TRAb negative.

### Thyroid Function Tests and Thyroid Autoantibody Measurement in Blood

Thyroid function tests (FT4 and/or FT3 plus TSH) were measured in blood just before each visit to each recruiting center. Methods and reference ranges are shown in Supplementary Table S1 (21). TRAbs were measured in an independent laboratory using the serum of each patient and the Cobas Roche for TRAK quantification (IU/L; cutoff > 0.3 IU/L) to obtain more comparable results across recruiting centers.

### DNA Extraction and 16S Ribosomal RNA Gene Sequencing Processing

Fecal samples were kept frozen at −20 °C for a maximum of 2 months before processing.

All samples were transported on dry ice to the same laboratory for identical processing as follows. Between 180 and 220 mg of slowly thawed feces at room temperature were individually placed in 2-mL FastPrep tubes prefilled with 0.1-mm silica spheres (FastPrep lysing matrix B, MP Biomedicals) and dissolved in 1-mL InhibitEX buffer (Qiagen Ltd). Nucleic acid extraction procedure followed the QiAmp Fast DNA Stool MiniKit (Qiagen Ltd), including 3 bead-beating steps. 16S rRNA gene sequencing of all samples was performed at Research and Testing RTL Genomics using primers for V1 to V2 regions of the 16S rRNA gene plus *Bifidobacteria* regions as previously used (11, 12) to generate 10 000 paired-end reads per sample per run on an Illumina MiSeq for a total of 2 sequencing runs.

Demultiplexed paired-end reads were checked for a preliminary quality control with FastQC and stored in NCBI repository (22). A total of 13 056 151 reads were obtained after joining R1 and R2. Further processing, including filtering and estimation of diversity indices (α and β), was performed with the QIIME 1.9 pipeline (23). Filtering parameters were (i) a maximum of 3 consecutive low-quality base calls (Phred < 19) allowed; (ii) fraction of consecutive high-quality base calls (Phred > 19) in a read over total read length greater than or equal to 0.75; (iii) no “N”-labeled bases (missing/uncalled) allowed. A total of 23 436 sequences were removed after quality filtering, and 13 032 715 sequences (99.8% average retention rate: maximum 99.9%, minimum 95%) were combined into a single FASTA file and were aligned against the SILVA closed reference database release 123 (24). The initial number of operational taxonomic units (OTUs) identified was 10 426; after removing OTUs with fewer than 10 counts in at least 2 samples, 5649 distinct OTUs were left. Bias in library size possibly due to sampling or sequencing were removed by normalizing OTUs in each library with the cumulative sum scaling method (25). Filtered and normalized OTUs were collapsed into phylogenetic levels (from phylum to genus).

### Production of Fecal Water

Fecal water (FW) was obtained from frozen fecal samples. After defrosting overnight at 4 °C and homogenization, fecal material was weighed and added to 2 × volumes (v/w) of sterile phosphate-buffered saline followed by homogenization through a shaker. Fecal slurry was centrifuged at 18 000*g* for 30 minutes (Avanti J26XP, Beckmann Coulter). Supernatants were carefully decanted and pressed through sequential syringe filters (Acrodisc Sterile Syringe Filters with Supor Membrane, Pall Laboratory) with pore sizes of 0.8 µm, 0.45 µm and 0.2 µm. FW samples were stored at −20 °C until used.

### ^1^H Nuclear Magnetic Resonance Spectroscopy Data Processing and Analysis of Fecal Water Samples

Nuclear magnetic resonance (NMR) acquisition was conducted using a Bruker AVANCE III 600 spectrometer at 600.13 MHz operating at a temperature of 27 °C. The NMR pulse sequence [recycle delay -90°-t_1_-90°-t_m_-90°-free induction decay acquisition] was used. An irradiation was used to suppress the water peak during recycle delay (4 seconds) and a mixing time (t_m_) of 100 ms. For each FW sample, 32 scans were accumulated into 64K data points with a spectral width of 20 ppm. Line broadening of 0.3 Hz was used before Fourier transformation of free induction decays. The assignment of the peaks was carried out using statistical total correlation spectroscopy (STOCSY) (26) in MATLAB and published spectral databases (27). The spectral phase and baseline were corrected and referenced to the TSP peak at δ^1^H 0.0 using Topspin (version 3.6). The spectral data were imported into MATLAB (version R2018a, MathWorks Inc) with a resolution of 0.0005 ppm. Regions including the TSP peak (δ^1^H −0.02 to 0.02), water regions (δ^1^H 4.65-5.10) were cut out. Remaining spectra were aligned due to peak shifts likely caused by different pH of the samples and probabilistic quotient normalization (28) was also applied.

### Statistical Analysis

Data handling and statistical analyses were performed with the R environment for statistical computing (29) unless otherwise specified. To estimate differences in within-sample α diversity indexes (ie, observed OTUs, Chao1, Shannon, and equitability) across nations of recruitment, a linear model with nations as a categorical fixed effect was used. When testing differences in α diversity indices among disease diagnoses (GD, GO, and HCs), thyroid status (hyperthyroid, euthyroid patients, euthyroid controls) and GO severity (no sign, GO mild, and moderate-severe), the linear model considered those as fixed effects (one per each model) and was designed to correct for nation, age, sex, and smoking habits. Between-sample β diversity was calculated from the Bray-Curtis dissimilarity matrix and was represented using nonmetric dimensional scaling (NMDS) with the Vegan R package (30). Differences in β diversity among samples for either nation or thyroid status were assessed using the permutational analysis of variance (PERMANOVA) implemented in the Adonis function (31) with 999 permutations. The model testing for thyroid status included the nation of provenance as a stratifying variable.

Similarly to the α diversity, differences in the normalized taxonomic counts at phylum and genus levels were estimated using linear regression models. The disease diagnosis (GD, GO, and HCs) or GO categories (no sign, GO mild, and moderate-severe) were considered as fixed effects and were corrected for thyroid status, nation, age, sex, and smoking habit. When looking at the thyroid status as a fixed effect, correction was performed on the basis of nation, age, sex, and smoking habit. α Indices and phylum/genus counts that were significantly associated with the fixed effects in each model were tested by pairwise *t* test comparisons (ie, between groups), and *P* values were corrected for multiple testing according to the Benjamini-Hochberg (BH) method (ie, false discovery rate, FDR) (32). Within this work, an adjusted threshold of *P* less than .05 was considered statistically significant.

Random forest (RF) (33) models were trained to predict to which diagnoses (control, GD, or GO) or GO severity (no sign, GO mild, GO moderate-severe) each sample belonged, based on the gut microbiota composition at the genus level. Both models accounted for the thyroid status, nation, age, and sex. An independent RF model was implemented to predict thyroid status (hyperthyroid, euthyroid patients, and euthyroid controls) irrespective of the initial diagnosis. The predicting variables included the genus level, age, sex, and nation. Samples with missing values for one of the aforementioned variables were excluded from the analysis. Cumulative sum scaling–normalized and filtered genus relative abundances with nonzero values in at least 20% samples were retained, scaled, and centered. The accuracy of the prediction was estimated through a 10-fold cross-validation arrangement repeated 3 times. The tuning hyperparameter *mtry* (number of variables [OTUs] included in the model), approximated as the square root of the number of columns of the data set, was tuned for values from 10 to 50, with either 5000 or 10 000 trees (ntree), using the R package Caret. RF was next run using the identified hyperparameters in each model providing the highest prediction accuracy from cross-validation to assign samples to groups, using the R package randomForest. The mean decrease accuracy was used for variable importance selection (ie, predictors driving the classification). Mean decrease accuracy was computed from permuting OOB (out-of-bag) data; for each tree, the prediction error on the OOB portion of the data was recorded, then the same was performed after permuting each predictor variable. The differences between the 2 errors are averaged over all trees and normalized by the SD of the differences.

Correlations between the gut microbiota biomarkers identified from RF variable importance, thyroid function tests (TSH and FT4 levels), and the TRAb thyroid-autoantibodies were assessed through the Pearson product-moment correlation coefficient (*r*) in R.

Differences in the genus normalized counts between TRAb-negative and TRAb-positive patients were calculated using a regression model per each time point (whether T0 or T1), correcting for thyroid status, nation, age, and sex. Genera differences between time points in each TRAb group (ie, positive or negative) were calculated using a paired *t* test with BH correction.

The processed spectral data from NMR spectroscopy of FW samples were analyzed using a set of multivariate statistical methods: principal component analysis and orthogonal projection to latent structure discriminant analysis (O-PLS-DA) in SIMCA-*P* + version 15.0 (Umetrics). Cross-validated analysis of variance was applied to evaluate the robustness of the O-PLS-DA models and *P* less than .05 was considered statistically significant. The main parameters calculated by these models are R^2^X and Q^2^Y, reflecting explained percentages of variation in the X-matrix (NMR data) and predictability of the model, respectively. To compare metabolites between 2 groups, univariate statistical analysis was carried out using a *t* test, followed by BH correction with statistical significance set at *P* less than .05.

## Results

### INDIGO Cohort and Gut Microbiota Differences Across Recruiting Centers

From the initially recruited 257 individuals, 105 GD/GO patients and 41 HCs provided fecal samples at the enrollment phase, based on the selection criteria (Supplementary Fig. S1A (21)), and were included in the microbiome study. Characteristics of patients providing samples included in the study are summarized in Table 1.

**Table 1.** Characteristics of 59 patients with Graves disease, 46 patients with Graves orbitopathy, and 41 healthy controls at recruitment

	Controls	GD	GO
No. of samples	n = 41	n = 59	n = 46
**Demographic**			
Age (mean, SD), y	46.08 (13.45)	46.39 (14.38)	47.04 (11.43)
Female (%)	32 (78.05%)	53 (89.83%)	40 (86.96)
White (%)	41 (100%)	50 (84.75%)	38 (82.60%)
Current smokers (%)	6 (14.63%)	8 (13.56%)	15 (32.60%)
**Thyroid status**			
Hyperthyroid (%)	0 (0%)	54 (91.53%)	35 (76.09%)
Hypothyroid (%)	0 (0%)	1 (1.65%)	0 (0%)
Euthyroid (%)	41 (100%)	4 (6.77%)	11 (23.91%)
**Orbitopathy status**			
No sign (%)	41 (100%)	59 (100%)	0 (0%)
Mild (%)	—	—	36 (78.26%)
Moderate-severe (%)	—	—	10 (21.74%)

Demographic features include age (mean, SD), sex, ethnicity (as a percentage), and current smokers (as a percentage); thyroid status indicates whether individuals were hyperthyroid (thyrotropin decreased, free thyroxine and/or free tri-iodothyronine increased relative to local reference ranges) or euthyroid at recruitment (the one hypothyroid case was due to antithyroid drug therapy and was excluded from analysis); presence and severity of GO defined as “mild,” “moderate-severe,” or “sight-threatening” eye disease, based on EUGOGO guidelines (20). Age is provided as the mean number of years and SD; all other characteristics are given as number and percentage.

Abbreviations: EUGOGO, European Group on Graves’ Orbitopathy; GD, Graves disease; GO, Graves orbitopathy.

Geographical variations of the gut microbiota have been previously reported, mostly associated with type of diet and lifestyle (34, 35). Analysis of α-diversity on the entire cohort (cases and controls) showed differences between Germany and the United Kingdom in the Shannon index of microbiome diversity (*P* = .007) and in the equitability index of microbiome evenness (*P* = .006) (Supplementary Fig. S1B (21)), while β-diversity indices revealed differences in terms of overall gut microbiota composition across geographic origin (Supplementary Fig. S1C (21)). At the phylum taxonomic level (Supplementary Fig. S1D (21)), *Firmicutes* were enriched in the Belgian cohort compared both to Italy (*P* = .024) and the United Kingdom (*P* = .038). *Proteobacteria* were enriched in UK samples compared to Germany (*P* = .03).

### Cross-sectional Analyses

#### Association between gut microbiota and autoimmune thyroid disease

**Figure 1.**
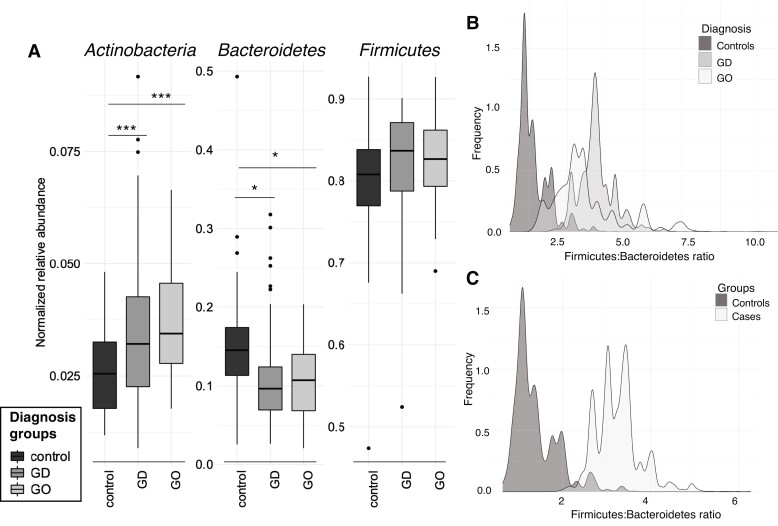
Gut microbiota composition in Graves disease (GD) patients and healthy controls at recruitment (T0). A, Distributions of the main phyla *Bacteroidetes*, *Firmicutes*, and *Actinobacteria* in controls (HCs), GD, and Graves orbitopathy (GO) patients. *P* values are generated from a pairwise *t* test with Benjamini-Hochberg (FDR) correction. Bootstrapped distribution of the *Firmicutes* to *Bacteroidetes* (F:B) ratio from 500 replicates of the data set for B, diagnosis groups (controls, GO and GD) and C, controls vs cases (GD + GO samples).

We compared the gut microbiota composition at recruitment among GD, GO, and HCs in a cross-sectional manner. The overall composition of the microbial community was similar across disease diagnoses (Fig. 1A); the *Actinobacteria* phylum increased in GD (*P* = .0017) and GO (*P* = .0001) compared to controls and were significantly more abundant in GO than in GD. In contrast, *Bacteroidetes* decreased in GD (*P* = .019) and GO (*P* = .019) compared to HCs. Interestingly, the *Firmicutes* to *Bacteroidetes* (F:B) ratio was significantly increased in GD vs HC (Fig. 1B) and in all cases (GD and GO) vs HCs (Fig. 1C). At a lower taxonomic level, 15 genera displayed significant differential abundance among GD/GO diagnoses and controls, resembling what was previously observed at the phylum level. Among others (Supplementary Fig. S2 (21)), *Bacteroides* spp were significantly decreased in GD (*P* = .018) and GO (*P* = .009) compared to HCs, while *Fusicatenibacter* spp was enriched in GD and GO compared to HCs (*P* = .013 and *P* = .002 respectively).

#### Association between gut microbiota and severity of Graves orbitopathy

The severity of GO was assessed in each recruitment center according to the EUGOGO guidelines (20). Of 46 GO patients, 36 had mild-active and 10 moderate-severe eye disease at recruitment (see Table 1). Sight-threatening GO patients were not included in this analysis and their gut microbiota composition was analyzed for the fecal material transplant in BALB/c mice (11).

**Figure 2.**
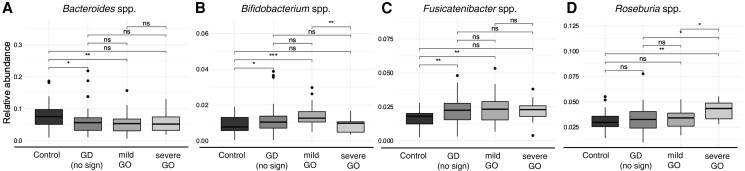
Significantly different genera across eye-disease severity (distributed as control, no sign/Graves disease [GD] only, mild, moderate-severe according to the EUGOGO guidelines). A, *Bacteroides* spp; B, *Bifidobacterium* spp; C, *Fusicatenibacter* spp, and D, *Roseburia* spp are shown. *P* values are generated from a pairwise *t* test with Benjamini-Hochberg (FDR) correction: *P less than .05; **P less than .01, and ***P less than .001.

At the genus level, reduced *Bacteroides* spp (*P* = .003; Fig. 2A), increased *Bifidobacterium* spp (*P* < .001; Fig. 2B), and increased *Fusicatenibacter* spp (*P* = .002; Fig. 2C) were significantly associated with mild GO, but not with moderate-severe GO. *Bifidobacterium* spp decreased in moderate-severe GO compared to mild GO (*P* = .032). On the other hand, *Roseburia* spp was enriched in moderate-severe GO compared both to HCs (*P* = .003), GD (*P* = .007), and mild GO (*P* = .022; Fig. 2D).

#### Gut microbiota composition influences metabolite composition in Graves orbitopathy

**Figure 3.**
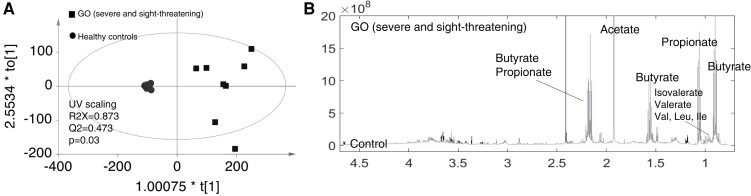
Analysis of fecal water metabolites associated with Graves orbitopathy (GO). A, Orthogonal projection to latent structure discriminant analysis of the fecal metabolites significantly discriminate between GO patients (both severe and sight-threatening; n = 10) and healthy controls (n = 11). B, GO fecal metabolites were enriched in short-chain fatty acids (butyrate, propionate, etc) compared to those of healthy controls.

We conducted NMR spectroscopy and orthogonal projection to latent structure discriminant analysis (OPLS-DA) of metabolites in FWs prepared from baseline samples from 11 HCs, 26 GD, and 10 GO patients. Principal component analysis revealed no clear separation of GD, GO, and controls (data not shown). However, OPLS-DA was able to discriminate between GO patients and HCs and indicated enrichment of several short-chain fatty acids (SCFAs; butyrate, propionate etc) in GO samples (Fig. 3).

#### Prediction of diagnosis based on gut microbiota composition

**Figure 4.**
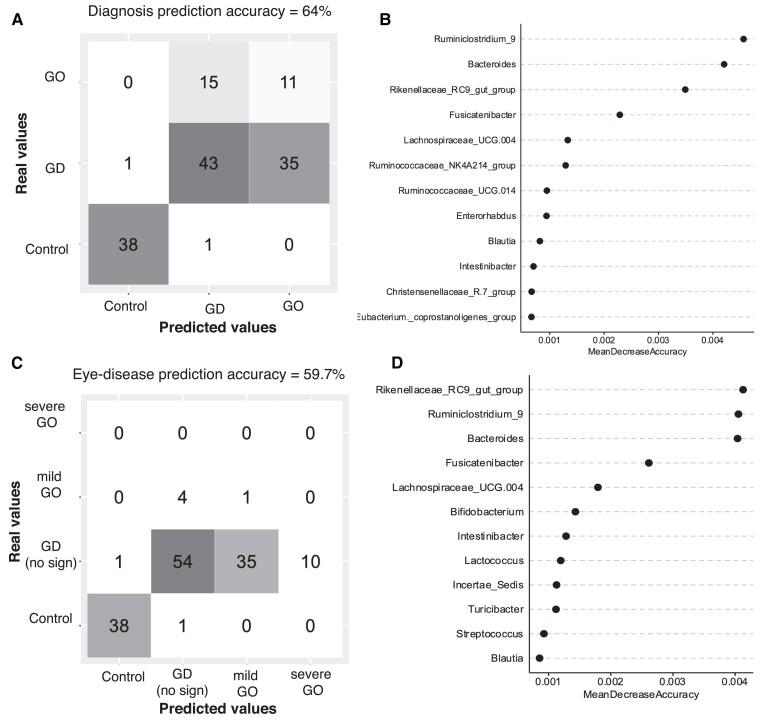
Random forest (RF) prediction of diagnosis based on the gut microbiome at genus level. A, Confusion matrix with the per-class classification for diagnosis (Graves disease [GD], Graves orbitopathy [GO], and healthy controls). Columns represent the true classification while the rows represent the predicted classification. B, Top-12 variable importance for diagnosis classification according to the mean decrease accuracy. The model included the thyroid status, nation of provenance, age, and sex as predicting variables. Thyroid status and nation of provenance were also identified among the most important variables. C, Confusion matrix with the per-class classification of the eye disease (no signs, mild, moderate-severe compared to healthy controls) and D, top-12 variable importance for eye disease classification according to the mean decrease accuracy.

We used the gut microbiota composition to either predict GD/GO/HC diagnosis or eye disease severity with the RF classification algorithm. RF prediction of the diagnosis returned a 64% accuracy across classes (overall OOB error rate = 36.11%). The highest prediction accuracy was found in the HC group (97.5% within-class accuracy), in which all but one sample (38/39) were correctly predicted as controls, followed by GD patients (nearly 73% within-class accuracy) with 43/59 samples correctly predicted as GD patients (Fig. 4A).

Prediction of eye disease severity was less accurate, with a 59% accuracy across classes (OOB error rate = 40.3%). The majority of the mild GO samples and all the moderate-severe GO were wrongly predicted as GD, showing a within-class accuracy of 8.34% and 0%, respectively (Fig. 4C). Variable importance rankings were obtained from the RF models based on mean decrease accuracy (Fig. 4B and 4D). Of note, *Bacteroides* spp was present in both classification models and represented one of the top bacterial biomarkers when predicting GO severity.

#### Association between gut microbiota and thyroid status

We next investigated the gut microbiota composition in euthyroid and hyperthyroid individuals regardless of the presence or absence of GO and compared with that of euthyroid HCs (as per inclusion criteria). One patient was hypothyroid due to ATD therapy and was excluded from the statistical analysis. The NMDS based on Bray-Curtis dissimilarity matrix showed a significant separation among groups overall (*P* = .02, based on 999 permutations; Supplementary Fig. S3A (21)). A significantly higher F:B ratio was observed in patients with hyperthyroidism compared with euthyroid patients (Supplementary Fig. S3B (21)). At the genus levels, *Fusicatenibacter* spp was significantly increased in hyperthyroid patients compared to euthyroid controls (*P* < .0001), while higher counts were found in euthyroid patients compared to controls, although these were not statistically significant (Supplementary Fig. S3C (21)). *Bacteroides* spp were significantly reduced in hyperthyroid patients compared to euthyroid controls (*P* = .0001), while there were no significant differences between euthyroid patients and euthyroid controls—although the euthyroid patients had reduced *Bacteroides* spp (Supplementary Fig. S3D (21)). The RF classification of thyroid status was less accurate than that of the initial diagnosis, with an overall accuracy of 60.14%. While hyperthyroid patients were mostly correctly predicted (99% within-class accuracy), most of the euthyroid controls and all the euthyroid patients were wrongly classified as hyperthyroid samples (Supplementary Fig. S3E and S3F (21)).

#### Correlation between gut microbiota, thyroid function, and autoantibody levels

**Figure 5.**
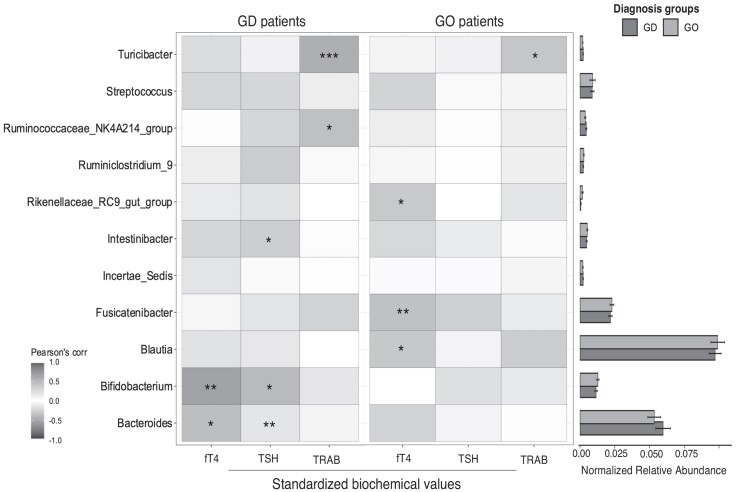
Correlations between thyroid function and the Graves' disease (GD) or Graves' orbitopathy (GO) gut biomarkers. Pearson's correlation coefficient was used to correlate the relative abundances of the selected bacterial biomarkers to the standardized levels of free-thyroxine (fT4), thyroid-stimulating hormone (TSH) and TRAB) in either GD or GO patients. All correlation coefficients are shown. Significant correlations are shown according to t-test *P*-value: * *P*<0.05; ** *P*<0.01 and ***P<0.001. Mean and standard deviation of each bacterial biomarker was shown for GD and GO groups.

Correlation between the gut microbiota biomarkers identified through the RF analysis and the levels of TSH and FT4 quantified in the blood was assessed using the Pearson product-moment correlation coefficient. Among biomarkers, *Bacteroides* and *Bifidobacterium* spp showed a significant correlation both with TSH and FT4 levels in the GD group. In particular, *Bacteroides* spp showed a positive correlation with TSH, although the majority of the GD patients—as per definition of GD—had low or undetectable TSH levels, and a weak negative correlation with FT4 levels (Fig. 5). *Bifidobacterium* spp showed a positive strong correlation with FT4 and a negative correlation with TSH. In GO patients only, *Blautia*, *Fuscatenibacter*, and *Rikenellaceae* spp showed positive correlations with FT4 levels. In the GD cohort, counts of the *Firmicutes* genus *Turicibacter* showed a weak negative correlation with TRAb levels while GO patients showed positive correlations instead, potentially sustained by the fact that GO patients tend to have higher TRAb levels, as there were no significant differences in the *Turicibacter* spp relative abundances between GD and GO.

#### Association between sex, smoking and Graves disease/Graves orbitopathy gut microbiota composition

Differences in the gut microbiota between sexes were also investigated within the GD and GO groups. Interestingly, none of the sex-associated genera showed a significant association with either disease diagnosis, or eye disease severity, apart from *Prevotella*-6 spp, which was also associated with thyroid status (Supplementary Fig. S4A (21)).

Cigarette smoking can also alter the composition of the gut microbiota (36, 37). However, smoking has long been considered a strong risk factor for GO (38). Among current, ex- and nonsmokers, 17 genera were differentially abundant within the HC group, 7 in GD, and 21 in GO (Supplementary Fig. S4A (21)). None of the smoking-associated genera in GD patients showed any similarities with those diagnosis-associated genera previously identified. Only *Bacteroides* spp, previously associated with diagnosis, eye disease severity, and thyroid status, showed a statistically significant association with smoking in GO patients. In particular, *Bacteroides* spp decreased in current smokers and ex-smokers, compared to never-smokers among GO patients (*P* = .024) (Supplementary Fig. S4B (21)).

### Longitudinal Analysis

#### Association between gut microbiota and response to antithyroid drug treatment

**Figure 6.**
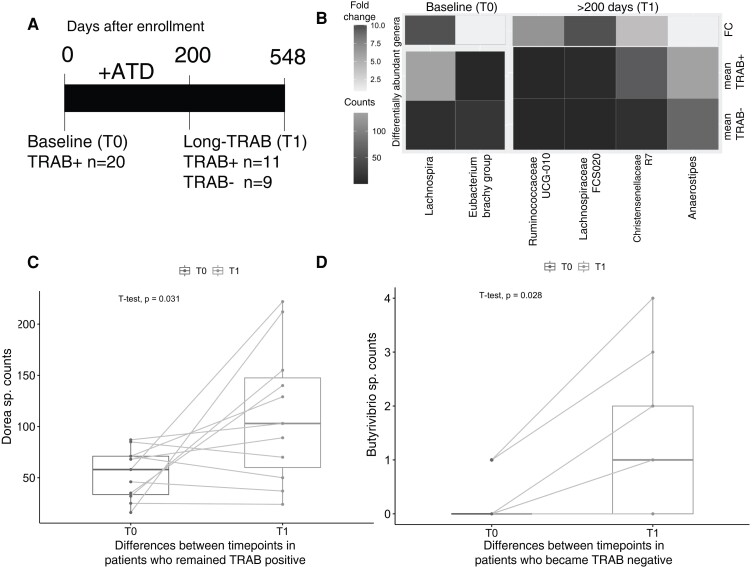
Differences in gut microbiota associated with thyroid autoantibody levels (TRAb). A, Rationale of the longitudinal study. N = 20 patients enrolled at baseline (T0; all TRAb positive per diagnosis) provided a follow-up fecal sample between 218 and 644 days (median 471 days) and here referred to as “200+ days” (T1). At follow-up (T1), 11 of 20 patients remained TRAb positive while 9 of 20 became TRAb negative. Long-TRAb refers to TRAb measurements taken at 200+ days. B, Heat map of the mean values of differentially abundant genera between TRAB-positive and TRAB-negative patients either at baseline (T0) or at T1. Fold change was calculated as the (mean TRAb positive – mean TRAb negative)/mean TRAb-negative values. C, Paired differences of *Dorea* sp between T0 and T1 in those patients who remained TRAb positive (n = 11) and D, paired differences of *Butyrivibrio* sp between T0 and T1 in those patients who became TRAb negative (n = 9). Paired *t* test corrected for Benjamini-Hochberg multiple tests.

We performed a longitudinal study on 20 of the initially enrolled patients who provided a follow-up sample after a minimum of 200 days from baseline (218-644 days, median 471 days from enrollment, T0) to ensure sufficient time to distinguish patients who responded to ATD, entered remission and became TRAb negative from those who remained TRAb positive and were more likely to relapse (39). At enrollment (T0), the 20 patients were all TRAb positive (per inclusion criteria), while at T1 (200+ days) 11 of 20 (55%) patients remained TRAb positive and 9 of 20 (45%) became TRAb negative (Fig. 6A). The baseline microbiome profile of patients who subsequently became TRAb negative differed from those who were persistently TRAb positive, with a significant excess of several members of the *Clostridiales* order of *Firmicutes*, *Lachnospira* sp, and *Eubacterium brachy* sp in the latter group (Fig. 6B). At T1, 4 different genera significantly increased in the TRAb-positive vs TRAb-negative groups. We next looked at differences between time points in those patients who remained TRAb positive; *Dorea* spp significantly increased in T1 vs T0 (paired *P* = .031; Fig. 6C). *Butyrivibrio* spp (ie, a butyric acid SCFA producer) significantly increased between time points in patients who became TRAb negative (Fig. 6D). Both *Dorea* and *Butyrivibrio* are also members of the *Clostridiales*.

By the completion of the study, 2 patients enrolled as GD had subsequently developed GO at Cardiff University Hospital (UK), referred here to as “GD to GO transition.” In particular, patient 1004, included in the study at the first visit October 3, 2014, as untreated and first diagnosis of GD, developed mild GO when euthyroid after 2 months (December 12, 2014). Patient 1013 had been enrolled as a relapsed GD October 31, 2014, and developed GO when euthyroid after about 3 months (January 30, 2015) (Supplementary Fig. S6A (21)). Some genera previously associated with GO status were observed in the within-patient GD-to-GO transition, for example, increased *Bifidobacterium* spp, *Lachnospiraceae* spp, and *Clostridiaceae* counts (Supplementary Fig. 6B and C (21)). Increased *Roseburia* spp was significantly increased in the relapsed GD patient, whose GO was considered to be more severe (Supplementary Fig. 6C (21)). The *Bacteroides* spp counts dramatically decreased during the GD-to-GO transition and slightly increased at a third time point, but below levels seen before the onset of GO (Supplementary Fig. 6D (21)).

### Defining a Microbiome Signature for Graves Disease

**Figure 7.**
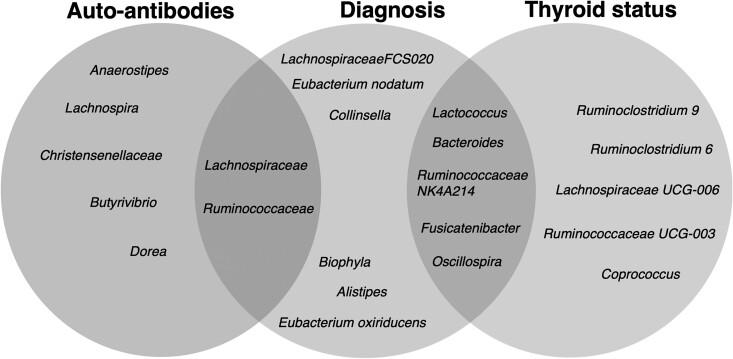
The INDIGO cohort gut microbiota signature attempted to identify operational taxonomic units (OTUs; out of necessity this covers all taxonomic levels from species to family) associated with disease status, autoantibody titers (TRAb), and free thyroxine (fT4) levels. We listed differentially abundant OTUs (from the discriminant analysis models) and/or important variables (from the random forest models) associated with TRAb, diagnosis (Graves disease [GD], Graves orbitopathy [GO], or controls), and thyroid status (whether hyperthyroid or euthyroid). We noted that all genera that associated uniquely with TRAb were *Firmicutes* of the *Clostridiales* family (the human equivalent of segmented filamentous bacteria implicated in several autoimmune conditions) (3). Similarly, genera uniquely associated with thyroid status were predominantly *Clostridiales*. In contrast, genera uniquely associated with disease status coincided predominantly with changes in genera from the *Bacteroidetes*, *Proteobacteria*, and *Actinobacteria* phyla. Intersections of the Venn plots represent the common significant genera between groups. Clostridiales are common both to TRAB and disease status and predominate in the overlap between thyroid and disease status along with members of the *Bacteroidetes* and *Bacillota* phyla. There was no significant overlap between autoantibodies analysis and thyroid status analysis. Owing to the nature of the experimental/sequencing procedure and analyses, this analysis is not intended as a mechanistic explanation of the microbiota role in the outcome of GD/GO.

The microbiota composition is dependent on a range of environmental factors, including diet, alcohol intake, stress levels, and smoking. Any changes observed between HCs and patients with GD and GO in our cohort could be due to additional variables such as thyroid hormone level and/or the autoimmune response to the TSHR. We attempted to identify the disease-specific species associated with each of these factors and any overlap, as illustrated in Fig. 7.

## Discussion

The increase in prevalence of all autoimmune conditions in recent decades (40–42) cannot be explained by changes in genetics, thereby highlighting the contribution of environmental factors such as diet, sex, smoking, radiation, chemicals, and drugs. Our earlier experience with induced animal models of GD/GO, which could not be replicated in different laboratories (43), led us to suggest a contribution from the gut microbiome in modifying the autoimmune response. Gut microbiota composition varies with diet and has been reported to influence a range of autoimmune diseases in humans and in animal models (44). Indeed, our more recent studies, which employed an improved robust model of GD/GO, identified disease-associated taxa. Furthermore, modulation of the mouse gut microbiome, before disease induction, had profound effects with antibiotic treatment reducing disease incidence and severity while human fecal material transfer increased both, thereby confirming its role. The present work aimed to assess the components of the gut microbiome in GD patients, with and without GO of varying severity, and compare it with HCs.

We enrolled individuals from 8 tertiary medical centers in 4 European countries; appropriate samples were collected, at diagnosis and at 2 time points following ATD treatment, for 16S rRNA gene sequencing, measurement of thyroid function and TRAb, and clinical evaluation of GO. Main findings included (i) an increased F:B ratio in GD/GO patients at diagnosis, as we observed in mice with induced disease; (ii) *Bacteroides* spp emerged as an important GO biomarker and was significantly associated with smoking in GO but not in GD; and (iii) GD patients who remained TRAb positive after at least 200 days of ATD treatment had significantly higher counts of the order *Clostridiales* than those who fully responded to treatment and became TRAb negative.

Our results differ from those reported in other studies, which have mainly been performed in Asia. Ishaq and colleagues (16) reported reduced *Bacteroidetes* in patients compared with controls, but this did not reach statistical significance; a similar cross-sectional study by Shi et al (17) compared 33 GO patients with 32 healthy controls and reported reduced diversity and significantly increased *Bacteroidetes* in patients’ gut microbiota. Subsequently TRAbs and gut microbiota were studied in the hyperthyroid patients at a single time point from the same group, revealing a significant positive correlation between TRAb and *Prevotella copri* and the *Bacteroides* family (45). These baseline results differ from ours, in which TRAb correlated positively with *Firmicutes* in GO patients. Differences in the proportions of men and women studied may provide some explanation. Shi and colleagues (46) then expanded their study to include GD patients and reported statistically significant differences between GD and GO at the phylum and genus level. An impressive study by Su and colleagues (9) demonstrated a possible lipopolysaccharide (LPS)-based mechanism to explain how the gut profile in GD could affect the Treg/Th17 balance. However, in line with other reports, many of their findings were based on small numbers since all analytes were not measured in all recruited individuals. More recently Jiang and colleagues (19) found increased *Bacteroidetes* and reduced *Firmicutes* in people with GD compared with matched controls—the exact opposite of that observed in our large, multicenter European cohort. The difference is puzzling but may reflect the importance of diet in defining the predominant enterotype in regions across the globe (47). A similar situation exists in rheumatoid arthritis, an autoimmune disease in which *Prevotella* spp have been reported to improve and suppress disease in different populations (48, 49). These apparent discrepancies highlight the difficulty in generalizing microbiome data across populations and are reminiscent of the heterogeneity in human leukocyte antigen–associated alleles, which predispose to autoimmune disease. As a fuller picture emerged, genetic susceptibility to GD was found to depend on geographic location and ethnicity, as illustrated by Lombardi and colleagues (50).

Aside from population differences, many of our patients had been started on ATD treatment by their community physician before attending the recruitment center, in contrast to most cases recruited in Asia. Little is known about the effect of ATD on the gut microbiota, other than work from Maier and colleagues (51), who assessed its effect on 40 selected bacterial strains *in vitro* and found minimal influence. All of our patients were hyperthyroid and TRAb positive when the baseline sample for 16S rRNA gene sequencing was provided and there were no statistically significant differences in α and β diversity indices when comparing clearly untreated (n = 35) with treated (n = 70) cases (data not shown). However, there were differences in abundance of several genera, including previously described bacterial biomarkers, between untreated and treated patients; for example, *Bilophila*, Ruminococcaceae UCG-010, and *Actinomyces* spp were decreased, but *Roseburia* spp was increased by ATD treatment vs untreated (Supplementary Fig. S5 (21)). It is possible that ATD explains the increase in *Roseburia* spp we observed in people with moderate-severe GO; they are butyrate-producing *Firmicutes*, which regulate many aspects of metabolism (52).

The importance of *Bacteroides* spp was underlined by its association with smoking in GO patients, who displayed further reduced counts, but not with GD patients in our cohort. Several reports have identified smoking as a major risk both for the development of GO and for poor response to treatment (53). A review of the effects of smoking on the gut microbiota by Savin et al (54) concluded that *Bacteroides*, *Clostridium*, and *Prevotella* spp were increased but *Bifidobacterium* and *Lactococcus* spp decreased in euthyroid patients. Although smoking cessation reversed the changes (37), other authors have reported that smoking induces permanent changes to the gut microbiome (55). Furthermore, we had the opportunity to analyze the microbiota in 2 GD patients who progressed to GO. In both cases the abundance of *Bacteroides* spp was further significantly decreased while *Bifidobacterium* spp, *Lachnospiraceae*, and Clostridiaceae increased (Supplementary Fig. S6 (21)).

*Bacteroides* spp have been highlighted in other autoimmune conditions, such as type 1 diabetes (T1D). *Bacteroides vulgatus* and, in particular, *Bacteroides dorei*, were increased in a Finnish cohort of children at high risk of T1D, a few months before seroconversion to having T1D autoantibodies (56). Elegant studies by Vatanen and colleagues (57) illustrated that biological outcomes depend on the interplay between bacterial species and their metabolites. They propose that the predominance of *B dorei*, whose LPS silences the immune system, explains the higher incidence of T1D in children in Finland and Estonia when compared with Russian infants, who are exposed to *E coli* LPS, a potent innate immune activator. Microbial metabolites also comprise SCFAs, which act via cytokines or G protein–coupled receptors to influence differentiation of Th17 and Treg cells (58). We conducted NMR and OPLS-DA of metabolites from FWs prepared from baseline samples; they were able to discriminate between GO patients and HCs and indicated enrichment of SCFAs (eg, butyrate, propionate) in GO patients (Supplementary Fig. S5 (21)). In contrast to the reduced *Bacteroides* spp counts, we demonstrated an increase in several taxa. In particular, several species from the *Clostridiales* order were more abundant in GD patients, who were resistant to ATD and remained TRAb positive. Clostridial species are the closest human equivalent to segmented-filamentous bacteria, which were necessary for disease to develop in mice predisposed to multiple sclerosis. Segmented-filamentous bacteria promote generation of Th17 cells, most likely via toll-like receptors expressed on gut epithelium (3).

Apart from smoking, women are at greater risk of GD than men. Our analyses already corrected for sex as a covariate, but we subsequently focused on differences in the gut microbiota between women and men in the GD and GO groups. None of the differentially abundant genera identified was previously associated with either disease diagnosis or eye disease severity. GD and GO male patients showed an increase of *Prevotella* genera, similar to the reports of Mueller and collaborators (59) in 4 different European countries.

Current studies investigating the interplay between microorganisms and autoimmunity tend to focus on their effect on T-cell subset differentiation, whereas previous research was based on the concept of molecular mimicry. In GD, *Yersinia enterocolitica* (YE) was proposed based on (i) the relatively high prevalence of antibodies to YE in GD patients (60, 61), (ii) the presence of binding sites for TSH on the YE envelope, and (iii) the fact that antibodies against thyroid membranes have been shown to bind YE (60). Results from proteomics have identified cross-reactivity between TSAbs and the outer membrane protein F (OmpF) epitope of YE (62). Additionally, a bioinformatic study of YE outer membrane proteins suggested it contained epitopes that could stimulate an antibody response that cross-reacts with T-cell epitopes (63). However, we did not detect any YE counts using 16S rRNA gene sequencing in cases or controls.

Our work has several limitations. The fact that approximately 75% of our GD/GO patients had received ATD has already been commented on, but it should be noted that it enabled us to dissect the varying contributions of treatment, thyroid hormone, and the autoimmune process to the dysbiosis observed in GD/GO. Despite enrolling more than 250 participants, we did not receive all required samples and clinical data at baseline and thus were unable to undertake microbiota analyses on all participants. The problem was exacerbated at the euthyroid and end of follow-up time points, which were available only for patients recruited early in the study. However, our study remains the largest yet conducted, with the number of participants being greater than those of all other reports, even at later time points. The thyroid function tests conducted at the different recruiting centers did not always include FT3, hence our correlations have used FT4. Our analyses were conducted at the genus level rather than individual species. The international, multicenter design of the study introduced problems inherent to heterogeneity from differing diets, multiple ethnicities, etc. However, the fact that we obtained similar changes in the gut microbiota in a heterogeneous, outbred human population with spontaneous GD to that which we described in inbred mice, fed an identical diet with induced GD, supports their validity.

We have summarized the INDIGO microbiome signature and attempted to identify OTUs (of necessity this covers all taxonomic levels from species to family) associated with disease status, TRAb titers, and FT4 levels (Fig. 7). We noted that all the genera linked with TRAb were *Firmicutes* of the *Clostridiales* family, while thyroid status coincided predominantly with changes in genera from the *Bacteroidetes*, *Proteobacteria*, and *Actinobacteria* phyla. To assess the microbiota components most relevant to GD and GO worldwide, future studies would benefit from collaborations between teams in Europe and Asia. Analyses should further include metagenomics (ie, at species and strain levels) and metabonomics (ie, microbial metabolites). Studies should also include a cohort of patients with nonautoimmune hyperthyroidism to unravel the interplay between the microbiome and thyroid hormones. Pairwise comparisons in individuals pre/post ATD treatment could identify beneficial modifications in the gut microbiota and whether they might be achieved by diet or other lifestyle changes.

In conclusion, we have demonstrated substantial perturbation of the gut microbiota in people with GD and its most severe form, GO. We have defined a microbiome signature (see Fig. 7) and identified the changes associated with the autoimmune process (TRAb) as distinct from those due to hyperthyroidism. Persistence of TRAb beyond 200 days of ATD treatment is predictive of relapse of thyrotoxicosis. If such patients can be identified at the time of diagnosis from their microbiome, this would be clinically very helpful and may alter management, let alone the implications of potential interventions to eradicate *Clostridiales*.

## Data Availability

16S rRNA gene sequencing reads and related metadata generated in this work were submitted under the NCBI accession ID PRJNA847182 (22).

## Abbreviations

AITD: autoimmune thyroid disease
ATDs: antithyroid drugs
BH: Benjamini-Hochberg
EUGOGO: European Group on Graves’ Orbitopathy
F:B: *Firmicutes* to *Bacteroidetes* ratio
FT3: free triiodothyronine
FT4: free thyroxine
FW: fecal water
GD: Graves disease
GO: Graves orbitopathy
HC: healthy control
INDIGO: Investigation of Novel biomarkers and Definition of the role of the microbiome In Graves’ Orbitopathy
LPS: lipopolysaccharide
NMDS: nonmetric dimensional scaling
NMR: nuclear magnetic resonance
OOB: out of bag
O-PLS-DA: orthogonal projection to latent structure discriminant analysis
OTU: operational taxonomic unit
QIIME: quantitative insights into microbial ecology
RF: random forest
rRNA: ribosomal RNA
spp: subspecies
SCFA: short-chain fatty acid
T0: time at enrollment
T1D: type 1 diabetes
Th17: T-helper cell 17
TRAb: total TSHR antibodies
Treg: regulatory T cell
TSAbs: thyroid-stimulating antibodies
TSH: thyrotropin
TSHR: thyrotropin receptor
YE: *Yersinia enterocolitica.*
